# Allergic sensitisation in tuberculosis patients at the time of diagnosis and following chemotherapy

**DOI:** 10.1186/1471-2334-9-100

**Published:** 2009-06-26

**Authors:** Linda K Ellertsen, Dag G Storla, Lien M Diep, Karl A Brokstad, Harald G Wiker, Geir Hetland

**Affiliations:** 1 Department of Environmental Immunology, Division of Environmental Medicine, Norwegian Institute of Public Health, Oslo, Norway; 2 Department of International Health, Institute of General Practice and Community Medicine, University of Oslo, Norway; 3 Centre for Imported and Tropical Diseases, Ullevål University Hospital, Oslo, Norway; 4 Aker University Hospital, The Research Center, Oslo, Norway; 5 Broegelmann Research Laboratory, The Gade Institute, University of Bergen, Bergen, Norway; 6 Section of Microbiology and Immunology, The Gade Institute, University of Bergen, Bergen, Norway; 7 Department of Microbiology and Immunology, Haukeland University Hospital, Bergen, Norway; 8 Department of Immunology and Transfusion Medicine, Ullevål University Hospital, Oslo, Norway

## Abstract

**Background:**

It is still a matter of debate whether there is an association between infection with *Mycobacterium tuberculosis *(*M. tuberculosis*) and allergy. Previously, we have shown higher levels of specific IgE to different inhalant allergens and total IgE in tuberculosis (TB) patients compared to controls. The objectives of this study were to evaluate a possible change in allergic sensitisation after successful TB treatment and to confirm the finding of our previous study of enhanced allergic sensitisation in TB patients compared to controls in a more controlled setting. Additionally, we wanted to determine the cytokine profile in the same groups and finally to evaluate the association between the presence of Bacillus Calmette-Guérin vaccination (BCG) scar and allergic sensitisation among the controls.

**Methods:**

Sera were analysed for specific IgE to inhalant allergens (Phadiatop) and total IgE by the use of ImmunoCAP 1000 (Pharmacia Diagnostics). Thirteen different cytokines were also analysed in the sera by multiplex bead immunoassay (Luminex 100, Luminex Corporation), and clinical symptoms of allergy and BCG scar were reported in a questionnaire.

**Results:**

A reduction in levels of specific and total IgE were observed after successful TB treatment. TB patients also had higher levels of specific and total IgE compared to healthy controls. Both interleukin (IL)-6 and interferon (IFN)γ were higher in TB patients compared to healthy controls. The levels of IL-6 were reduced after successful TB treatment. The presence of a BCG scar was associated with a reduced risk of developing allergic sensitisation.

**Conclusion:**

We observed a reduced level of allergic sensitisation after successful TB treatment. TB patients seem to be more allergically sensitised than healthy controls, confirming our previous finding. Furthermore, we observed an inverse association between allergic sensitisation and visible BCG scar, which adds additional support to the hygiene hypothesis.

## Background

Infections with *M. tuberculosis *remain a major health problem around the world, leading to 2–3 million deaths annually. There are 7–8 million new cases of TB every year [1], and a third of the human population has been estimated to be infected by *M. tuberculosis*. While TB is a problem in developing countries, allergy has been an increasing problem in developed countries. In 1989 Strachan proposed the hygiene hypothesis, stating that an increase in allergy could be a result of reduced childhood infections [2]. The mechanistic rationale behind the hygiene hypothesis has been challenged. Theories of an imbalance between Th1/Th2 responses and reduced immune suppression by regulatory T cells have been discussed as alternative explanations [3,4]. Widespread attention has been given to the possible link between *M. tuberculosis *infection and allergy, in which *M. tuberculosis *infection has been suggested to promote a reduction in allergic disorders. Previously, increased levels of total IgE has been documented in TB patients compared to controls [5-8], and a decline in total IgE after TB treatment has been observed [5-7,9]. However, total IgE is a poor marker for allergy because it can also increase after parasitic infection. Specific IgE to different allergens correlates well with allergic disease. Despite the importance of specific IgE as a marker for allergic disease, limited and conflicting results have been reported on the association between specific IgE and TB [9,10]. The present work is an extension of a previous study, which showed an increase in allergic sensitisation among TB patients compared to healthy controls [8]. The main goals of this paper were to study whether the allergic sensitisation changed after TB treatment, and to confirm the finding of higher levels of allergic sensitisation in TB patients compared to more optimally selected controls matched by gender, age, and socio-economic status. Additionally, we wanted to compare the expression of cytokines between the groups. Finally, the association between allergic sensitisation and Bacillus Calmette-Guérin (BCG) vaccination scar was evaluated in healthy controls. To answer these questions, specific IgE antibodies to a range of inhalant allergens (Phadiatop), total IgE, and cytokine responses in the sera were analysed. Additionally, a questionnaire about allergy status was evaluated.

## Methods

### The study population

The Bangladesh Rural Advancement Committee (BRAC) organised the TB control programme in cooperation with the National TB Programme (NTB) [11]. The present study was a longitudinal study. Sera were collected from 108 TB patients at the time of diagnosis, prior to initiation of treatment, and from 216 healthy controls living in the Sunamganj district in Northern Bangladesh during 2004 and 2005. In addition, sera were collected from a subgroup of 71 TB patients after completed treatment. The Sunamganj district is an isolated rural area inhabited mainly by farmers. Collection of the sera was organised by a local consultant company, Destiny Associates, in cooperation with BRAC and the NTB. The patients were recruited among sputum smear positive (SS+) patients living in 4 upazillas (sub-districts) in Sunamganj (Chatak, Derai, Duarabazar and Sunamganj Sadhar). The controls were recruited by our field staff from the same villages as the patients and had no history of previous TB. The controls were recruited to match the TB patients on group level by gender, age, and socio-economic status. Socio-economic status was defined by landownership (landowner or landless), which type of housing (brick, tin-shed or cathca (non-permanent)), education (illiterate, primary, or secondary school), and occupation (three categories: day labourer/disabled/unemployed, student/housewife/worker (salary), and own-enterprise/farming own land). The four different variables were given a score and added for each participant; 0 was the lowest and 8 was the highest socio-economic status. Informed consent was obtained from the patients and healthy controls. Characterisations of the participants of the different groups are listed in Table 1. The controls and post-treatment patients were paid Tk 600 (about US $9.00) for each blood sample. The following patients were excluded from the comparison of patients before and after treatment: patients who did not want to give blood, patients who defaulted (quit the treatment before it was completed) or failed treatment (the treatment was not successful), or patients who died.

**Table 1:** Distribution and characterisation of the groups

	Controls	Tuberculosis patients	
		
		A: Before treatment	B: Subgroup; follow-up analyses
Number	216	108	71
Gender (%) (females, males)	33.5/66.5	33.3/66.7	27.8/72.2
Age, Mean ± SD	41.1 ± 15.5	40.1 ± 14.8	40.4 ± 15.0
Smoking, no/yes (%)	53.2/46.8	59.5/40.5	62.5/37.5
BCG scar, absent/present/unknown (%),	75.2/18.3/6.4	91/8.1/0.9	90.3/9.7/0
Reported allergy, n (%)	14 (6.4)	21 (18.9)	13 (18.1)
Weeks of TB symptoms Mean ± SD		10.5 ± 5.7	10.4 ± 5.9

BCG = Bacillus Calmette-Gu, rin, A: These patients were included in the analyses between tuberculosis patients and healthy controls. B: The subgroup consisted of 71 patients which were followed-up after treatment. These patients were included in paired-analyses concerning before and after tuberculosis treatment.

The blood samples were centrifuged and frozen locally before they were shipped on dry ice to Norway. The patients were interviewed by the field staff using a questionnaire for information about allergy (yes/no), smoking, socio-economic status, and BCG vaccination (reported as visible BCG scar or not). The project was approved by the regional Committee for Medical Research Ethics in Western Norway and the Committee for Medical Research Ethics of Bangladesh Medical Research Council (BMRC).

### Tuberculosis treatment regimen

The patients were treated according to the NTP standard regimen for new SS+ patients; 2 months with isoniazide, rifampicin, pyramizinamide, and ethambutol followed by 4 months of isoniazide and rifampicin.

### Specific and total IgE

Specific and total IgE were analysed by ImmunoCAP 1000 r system in 2006 and 2007. This is an automated allergy-testing system from Phadia AB (Uppsala, Sweden). The Phadiatopr was used to analyse specific IgE to a combination of different inhalant allergens (cat, dog, horse, timothy, birch, hazel, mugwort, wall pellitory, mold fungus (*cladosporium herbarium*), and house dust mite (*Dermatophagoides pteronyssinus, Der p1 and Der p2*)). We chose to use the European Phadiatop test on recommendation from the producer as the most relevant broad spectre allergy test containing several common allergens in Bangladesh. The test was performed according to the manufacturer's instructions, and the Phadiatop results are expressed in PAU/l (Phadia Arbitrary Units/l), while total IgE results are expressed in kU/l.

### Cytokines

Cytokines were analysed by multiplex bead immunoassay (Luminex 100, Luminex Corporation, Austin, TX, USA) according to the manufacturer's instructions and using kits from Biosurce (Cat. No.; LHC 0001, LHC 0151, LHC 9121, LHC 0171, Invitrogen, Carlsbad, CA, USA). The cytokines were quantified (pg/ml) by using standards supplied with the kits. The detection limit of the different cytokines were 3 pg/ml (IL-5, IL-6, IL-8), 5 pg/ml (IFNγ, IL-4, IL-10), 6 pg/ml (IL-2), 10 pg/ml (IL-12p70, IL-15, IL-17, tumour necrosis factor (TNF)α) and 15 pg/ml (Granulocyte-macrophage colony-stimulating factor (GM-CSF), IL-1β), as given by the manufacturer. More than 20% of the test sera in one of the groups had to be above the detection limit to be analysed in a statistical model.

### Statistics

There is a strong correlation between Phadiatop quantifications and the sum of the individually measured specific IgE antibodies to the allergens included in the Phadiatop assay [12,13]. The rate of respiratory allergic disease has been shown to increase linearly with the increase in specific IgE (Phadiatop) [13,14]. Consequently, we have analysed the results with a continuous scale instead of the negative/positive cut-off value as suggested by the manufacturer. Non-parametric tests were used to analyse specific and total IgE due to skew distributions of the data. The Mann-Whitney test for two independent groups was applied to compare TB patients with healthy controls, and differences between TB patients before and after treatment were analysed by Wilcoxon signed-rank test for paired data. The median with a 95% bootstrapped confidence interval for specific and total IgE values were estimated using the bootstrapped method [15] with 10,000 bootstrap replications.

To adjust for possible confounders when comparing healthy controls to TB patients, a multivariate logistic regression model was used. We wanted to estimate the association of TB and allergy using tuberculosis disease as the dependent variable. The independent variables in the multivariate logistic regression analyses were Phadiatop, reported allergy, BCG status, and smoking. Phadiatop and reported allergy were run separately with BCG status and smoking to avoid colinearity [16]. The Phadiatop values were stratified into class 0–2 (from 0 to 3.49 PAU/l) and class 3–6 (3.50 to 100 PAU/l) before including them in the logistic regression model. The study population was matched on group level by gender, age, and socio-economic status, and therefore not included in the analyses as confounders. In addition, the controls were analysed separately to look at the association between visible BCG scar and Phadiatop result. The control group was divided in two based on the Phadiatop values (class 0–2 and class 3–6) and used as the dependent variable in the logistic regression model. Gender, age, and socio-economic status, visible BCG scar and smoking were included in the model as independent variables, since no matching was performed at this level. The adjusted odds ratios (AOR) with 95% confidence intervals and two-sided p-values were given. These analyses were performed using the Statistical Package for Social Science (SPSS) for Windows, version 14.0.

The cytokine data had a skewed distribution and there were a high number of ties in the data. Therefore, we used a permutation test to estimate p-values for comparison of two independent groups to evaluate differences between TB patients before and after treatment [16-18]. A Bonferroni correction was used to correct for 9 multiple comparisons giving a statistical significance level of 0.05/9 = 0.0056. The statistical software R version 2.2.1 for windows was used to perform the permutations [18].

## Results

### Comparison of allergy markers among TB patients before and after treatment

**Figure 1:**
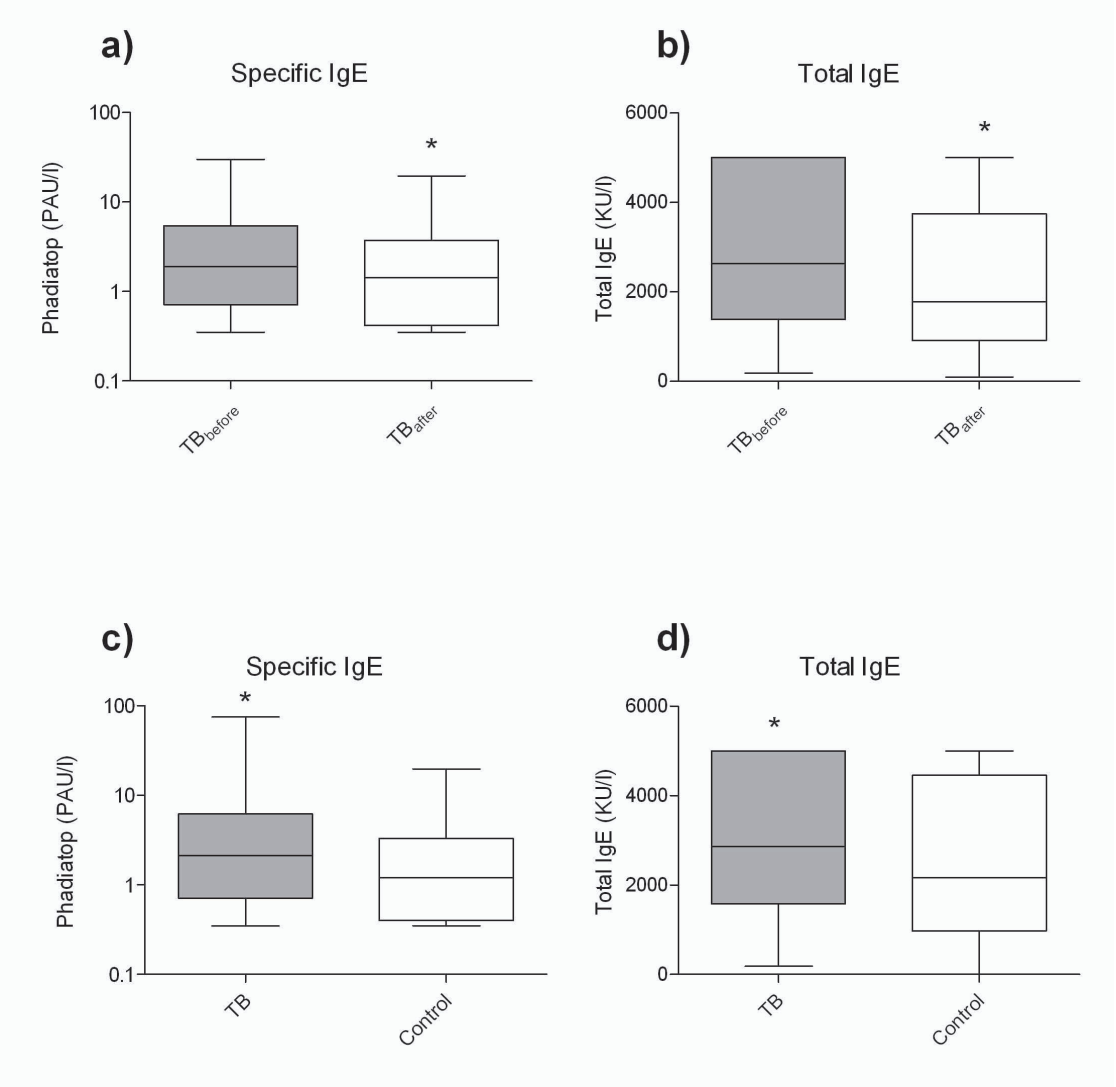
**Both specific (a) and total (b) IgE were reduced after successful treatment of TB patients**. Total (c) and specific (d) IgE were higher in TB patients compared with healthy controls. The box plot indicates the 25 percentile, the median and the 75 percentile. Error bars above and below the box illustrate the maximum and minimum values.

The levels of specific IgE (p = 0.0005) and total IgE (p = 0.0005) were significantly lower in TB patients after treatment compared to those before treatment (Fig. 1a and 1b). Analysis by gender revealed similar differences (Table 2). There was no significant difference between genders within each group.

**Table 2:** Comparison of tuberculosis patients before (TB_before_) and after (TB_after_) treatment (subgroup, n = 71).

Marker	Group	Median	95% bootstrapped CI	Significance p
Total IgE	TB_before_	2636	2078 – 3954	0.0005 *
(kU/l)	TB_after_	1781	1224 – 2525	
	TB_before _male	2587	1972 – 4058	
	TB_after _male	1730	1154 – 2651	0.0005 *
	TB_before _female	2697	1559 – 5000	
	TB_after _female	2285	925 – 3017	0.015 *
Phadiatop	TB_before_	1.89	1.10 – 2.79	
(PAU/l)	TB_after_	1.44	0.86 – 1.99	0.0005 *
	TB_before _male	1.89	1.03 – 2.94	
	TB_after _male	1.44	0.85 – 2.61	0.0005 *
	TB_before _female	1.97	0.70 – 3.98	
	TB_after _female	1.34	0.36 – 2.22	0.004 *

Wilcoxon Signed-rank Test

### Comparison of allergy markers among TB patients and controls

TB patients had significantly higher levels of both specific IgE (p = 0.001) and total IgE (p = 0.016) (Fig. 1c and 1d) compared to healthy controls. Separation of the groups by gender revealed similar differences for specific IgE, while the total IgE levels were not significantly different (Table 3). There were no gender differences within each group (data not shown). Allergy reported in the questionnaire was indicated by 18.9% of the TB patients and by 6.0% in the control group. To adjust for possible confounders, a multivariate logistic regression model was used to study the association between TB disease at diagnosis and allergy (Table 4). To increase the probability of clinical symptoms, we used a higher cut-off value than the cut-off value suggested by the manufacturer; negative (class 0)/positive (class 1 to 6). Phadiatop values were therefore stratified into class 0–2 (from 0 to 3.49 PAU/l) and class 3–6 (3.50 to100 PAU/l). However, we also found a significant difference using the cut-off value recommended by the manufacturer (< 0.35 = negative, > 0.35 = positive) (AOR = 1.96, CI 1.01 – 3.78, p = 0.046). In total, 60.2% of the TB group, and 76.8% of the control group were distributed to class 0–2, while 39.8% and 23.6%, respectively, were distributed to class 3–6. The adjusted logistic regression analyses revealed that allergy was positively associated with TB disease. Smoking had no effect on the prediction for TB. However, the presence of BCG scar was associated with reduced TB disease (Table 4).

**Table 3:** Comparison of TB patients before treatment (n = 108) and healthy control (n = 216).

Marker	Group	Median	95% bootstrapped CI	Significance p-value
Total IgE (kU/l)	Control	2160	1799 – 2835	0.016 *
	TB	2868	2514 – 4060	
	Control male	2710	1800 – 3422	0.121
	TB male	3050	2269 – 4098	
	Control female	1928	1427 – 2409	0.052
	TB female	2697	2150 – 5000	
Phadiatop	Control	1,21	0.92 – 1.51	0.001 *
(PAU/l)	TB	2,24	1.53 – 2.78	
	Control male	1.31	0.92 – 1.87	0.008 *
	TB male	1.98	1.18 – 3.30	
	Control female	1.02	0.65 – 1.37	0.044 *
	TB female	2.40	1.00 – 3.55	

MannWhitney

**Table 4:** The association of TB and allergy markers in TB patients before treatment (n = 108) as compared with healthy controls (n = 216).

	AOR (95%CI)	P-value
Phadiatop (Class 0–2/class 3–6)	2.19 (1.30 – 3.68)	0.003
BCG scar (not visible/visible)	0.45 (0.21 – 0.99)	0.046
Smoking (no/yes)	0.72 (0.43 – 1.18)	0.186

Reported allergy (no/yes)	3.27 (1.57 – 6.83)	0.002
BCG scar (not visible/visible)	0.38 (0.18 – 0.84)	0.017
Smoking (no/yes)	0.30 (0.47 – 1.26)	0.297

Multivariate logistic regression models were used to estimate adjusted odds ratio (AOR) for TB as the dependent variable. Either Phadiatop (ImmunoCAP class 0–2 and class 3–6) or reported allergies were included in the analyses as independent variables together with visible BCG scar and smoking.

### Cytokines

**Figure 2:**
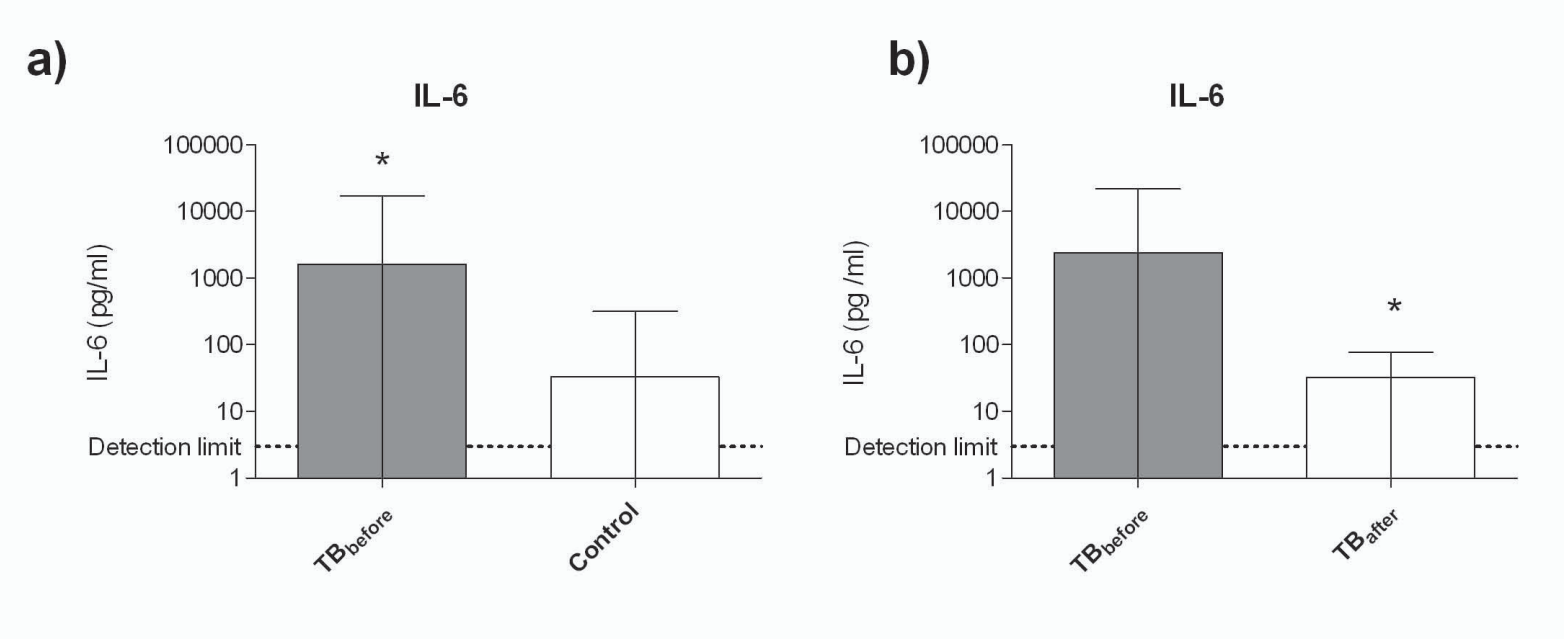
The levels of proinflammatory cytokine IL-6 were (a), significantly higher in TB patients compared with healthy controls (*p < 0.0001), and (b) significantly reduced in TB patients after treatment (TB_after_) compared with before started treatment (TB_after_). The IL-6 levels are given as means ± standard deviation (SD).

In general, the concentrations were low for most of the cytokines, except for IL-6 and IL-8. Significantly higher levels of IL-6 were found in TB patients at the time of diagnosis compared to controls (p < 0.0001) (Fig. 2a). A clear reduction was observed in IL-6 levels after TB treatment (p < 0.0001) (Fig. 2b). On the other hand, there was no significant difference in IL-8 concentrations in TB patients before treatment as compared with controls (p = 0.17) or in TB patients before and after TB treatment (p = 0.40). The levels of IFNγ were significantly higher in TB patients before treatment (mean ± SD: 9.1 pg/ml ± 10.8) compared with controls (mean ± SD: 6.7 pg/ml ± 17.9) (p < 0.0001). However, no significant difference was found in IFNγ concentration between TB patients before and after treatment (p = 0.10). There was no significant difference in the concentrations of GM-CSF, IL-1β, IL-2, TNFα, IL-12p70, and IL-15 between TB patients before treatment and controls, or between TB patients before and after treatment. The serum levels of IL-4, IL-5, IL-10 and IL-17 were below the detection limit of this system.

### Association between BCG and allergy markers in the control group

To investigate the effect of visible BCG scar alone, we also analysed the control group separately by categorizing the controls into two groups depending on the Phadiatop values (class 0–2 and class 3–6). In a logistic regression model, the presence of a BCG scar was associated with a reduced risk for developing allergic sensitisation (AOR = 0.32, CI 0.11 – 0.93, p = 0.037). Neither age, socio-economic status, gender, nor smoking had a statistically significant effect on the association between TB and BCG scar. No significant difference was observed using the cut-off value as suggested by the manufacture (< 0.35 = negative, > 0.35 = positive).

## Discussion

We observed reduced levels of IL-6, specific and total IgE after successful TB treatment. The levels of IFNγ, IL-6, specific and total IgE were higher in TB patients compared to healthy controls. Furthermore, an inverse association was found between the presence of a BCG scar and specific IgE.

The key finding of reduced levels of specific and total IgE after successful treatment, indicates a reduction in allergic sensitisation in TB patients after treatment for TB. To our knowledge no longitudinal studies analysing TB patients before and after treatment have been published previously on specific IgE using the Phadiatop test. There is a positive correlation between Phadiatop levels and allergy symptoms [13], and therefore it is possible that the observed difference is clinically relevant. In contrast to our study, Mungan *et al*. observed a lower percentage of positive Phadiatop test and skin prick test in TB patients with active disease compared to patients with a past history of TB [10]. However, the authors did not follow the same patients before and after treatment, and only 31 patients with a history of TB were analysed. Adams *et al*. [9] found no difference in specific IgE towards house dust mite, cockroach or Bermuda grass before and after TB treatment. The Phadiatop test includes a broader selection of allergens, and we have also included significantly more patients, which might explain the conflicting results. The finding of reduced level of total IgE after successful TB treatment is supported by several other studies [5-7,10].

Both specific and total IgE levels were higher in TB patients as compared to controls, confirming the findings of our previous study [8]. In contrast to our study, Adams *et al*. [9] found no difference in specific IgE among 23 TB patients and 33 healthy controls. The controls were highly improved in the present study by matching gender, age, and socio-economic status. After adjusting for smoking and the presence of BCG scar, the multivariate logistic regression model used showed a positive association between TB and the markers for allergy (specific IgE or reported allergy in the questionnaire). Whether individuals with allergy have an increased risk for developing TB, or TB patients have an increased risk for developing allergy is a difficult question. However, patients with active TB have distinct immunological features that are different from individuals with latent TB. For example, healthy individuals with latent TB have increased IL-4δ2 mRNA levels (an antagonist of IL-4) rather than increased IL-4 mRNA levels, as observed in progressive TB [19]. To our knowledge, the only prospective study performed on this issue followed 10 *M. tuberculosis *infected health-care workers for 5 years, out of which 6 developed TB. [20]. Prior to disease development, they had increased intracellular IL-4 and reduced IFNγ levels in their CD8^+ ^and γδ^+ ^T cell subsets in response to *M. tuberculosis *antigen stimulation. Although small, this study indicates a predisposition for a Th2 response. Only 5–10% of infected individuals develop TB disease, and therefore it is conceivable that those particular individuals might be genetically predisposed for a Th2 response.

There are some limitations to this study. We were not able to adjust for helminthic infection and HIV in our study, which both represent confounders. Specific IgE towards helminths and *M. tuberculosis *[21] itself could also partly explain the increased level of total IgE and the specificity of the total IgE. There could be a general polyclonal stimulation, which could explain the increased levels of IgE. A reduction of IgE and IL-6 after TB treatment might also be explained by a reduction of a polyclonal stimulation or the use of anti-tuberculosis drugs, but to our knowledge a beneficial effect by anti-tuberculosis drugs on IgE levels has not been reported. Another limitation to the study is the questionnaire-based diagnosis of allergic disorder. People in developing countries like Bangladesh may not be familiar with the term allergy, and it is possible that sick TB patients might report more symptoms than healthy individuals. Therefore, the results regarding the questionnaire should be read with caution. We tried to compensate for some of this limitation by having questionnaires filled in by the field staff.

Additionally, we examined the difference in the cytokine profile (Th1-, proinflammatory-, Th2-, and Treg cytokines) in TB patients and controls, and between TB patients before and after treatment. Significantly higher levels of the proinflammatory cytokine IL-6 and the Th1 cytokine IFNγ were observed in TB patients compared to controls. The levels of IL-6 were reduced upon successful TB treatment. Previous studies have also shown a higher level of IL-6 in serum from TB patients compared to controls [21,22], as well as a decrease after TB treatment [21]. Since IL-6 has been associated with protection against *M. tuberculosis*, it seems reasonable that IL-6 is elevated in patients with TB disease. However, the reduced levels of IL-6 after TB treatment might also be due to reduced allergic sensitisation. Previously, IFNγ has been found to be increased in serum from TB patients relative to controls, confirming our results [21-25]. In contrast to our observation, a decrease in IFNγ concentration during treatment has previously been found [22,26]. There are some limitations to the cytokine data, and thus a more complete cytokine profile with respect to Th1, Th2 and Treg cytokines could not be established. For some of the cytokines there were only some individuals with detectable levels and hence, comparison of the groups with respect to IL-4, IL-5, IL-10 and IL-17 could not be performed. Therefore, it is uncertain whether these cytokines were absent from the serum or too low for detection by the Luminex system. Some cytokines are sensitive to storage [27]. Variable clotting time, freeze-thaw cycles, and processing of the samples can also influence the degradation. These factors prohibited exact quantification of cytokines in the serum, but caution was taken to handle the samples equally, allowing us to compare the data from the different groups.

To investigate the effect of BCG scar alone (without TB disease), we also analysed the control group separately by categorising the controls into two groups depending on the Phadiatop values (class 0–2 and class 3–6). The presence of BCG scar was associated with a reduced risk of developing allergic sensitisation. This finding agrees with the idea above, and points to a similar anti-allergic effect of live *M. bovis*, BCG. If BCG vaccination protects against development of allergy, and there are more people in the TB group who are not BCG vaccinated, then this could partly explain the higher percentage of allergic sensitisation among TB patients. Previous studies that have investigated the effect of BCG vaccination on allergy, have been diverging, maybe due to reasons described elsewhere [19]. It is difficult to investigate the effect of BCG alone because the healthy controls have probably been exposed to mycobacteria, either *M. tuberculosis *or other environmental mycobacteria. Additionally, the participants in the study might have been vaccinated even though one could not observe a BCG scar. Therefore, the result regarding the protective effect of BCG scar should be read with caution.

To summarise, we observed a reduced level of allergic sensitisation after successful TB treatment, which may be due to down-regulation by dead or dying *M. tuberculosis *bacilli, a direct effect of anti-tuberculosis drugs, or elimination of a general stimulation by *M. tuberculosis*. TB patients seem to be more allergic sensitised than healthy controls confirming our previous finding of enhanced allergic sensitisation in TB patients. This may indicate that TB patients are more susceptible to develop allergy or that allergy patients are more disposed to develop tuberculosis upon infection with *M. tuberculosis*. The inverse association of allergic sensitisation and visible BCG scar adds additional support to the hygiene hypothesis, and might have implications for prevention of allergic diseases in the future.

## Conclusion

In the present study we observed a reduction of allergic sensitisation in TB patients after successful treatment. Additionally, a higher degree of allergic sensitisation in TB patients compared to healthy controls was found. Furthermore, we observed a negative association between allergy development and visible BCG scar.

## Competing interests

The authors declare that they have no competing interests.

## Authors' contributions

LKE participated in the designing of the study, collection of the sera, performing of the analyses, and interpretation of the data, and she drafted the manuscript. DGS participated in the collection of the sera, LMD advised and performed some of the most complicated statistical analyses, KAB performed the Luminex analyses, and GH and HGW participated in the design of the study, collection of the sera and revision of the manuscript. All co-authors have read and approved the manuscript.

## Pre-publication history

The pre-publication history for this paper can be accessed here:

http://www.biomedcentral.com/1471-2334/9/100/prepub
